# Strengthening the capacity of and professionalizing human resources for supply chain in Indonesia through the SCM Provincial Network

**DOI:** 10.1186/2052-3211-7-S1-O14

**Published:** 2014-12-17

**Authors:** Hidayati Mas’ud, Russell Vogel, Rio Chandra Dewa, Nani Sukasediati

**Affiliations:** 1 Pharmacy DirJen, Ministry of Health, Jakarta, Indonesia; 2 USAID|DELIVER PROJECT, John Snow Inc., Jakarta, Indonesia

## Background

Indonesia is a large archipelago consisting of 34 provinces spread over 17 key islands. Because of its geography, addressing discrepancies in HR for SCM capacity over the entire country is a challenge. To professionalize and strengthen the capacity of its HR for SCM, the MOH, in collaboration with the People that Deliver (PtD) initiative, the World Health Organization (WHO), and the USAID | DELIVER PROJECT, developed the Provincial SCM Network in 2011.

## Method

Stakeholder workshops to explore solutions to these challenges for HR for SCM resulted in the formation of the Provincial SCM Network. The goals of the Network are: capacity building of members, strengthening professionalism of supply chain managers, and development of the Network. Members include senior provincial SCM staff and/or province drug warehouse chiefs from provincial health offices. Members participate in national meetings and use an effective group communication system through the WHO Knowledge Gateway.

## Results

Five national network meetings have been held since 2011 (one every four to six months). The meetings have succeeded in building a true network of public health supply chain managers. For instance, to help senior SCM professionals better understand the SCM needs of the disease programs and to improve collaboration with program managers regarding their drug management needs, meetings have covered such topics as drug management information for HIV/AIDS, malaria, TB, maternal/child health, and nutrition programs. Network meetings also built member capacity in such skills as advocacy, effective communication, and using the WHO Knowledge Gateway.

## Discussion

In addition to providing opportunities for regular contact and effective networking among members, the Network has strengthened Indonesia’s commitment to building capacity and professionalism of HR for SCM. Participation in the Network has also galvanized provincial SCM leaders to continue their professional development. An ongoing challenge facing the Network is the cost and time needed to organize and attend meetings. While looking for more sustainable approaches, the MOH and its partners will continue and expand the program by using committed Network members to further strengthen SCM systems and act as agents of change for continued professional development.

## Lessons learned

The Provincial SCM Network has proven to be an effective means to build SCM capacity and encourage professionalism among SCM staff. There is great interest in the Network by SCM staff because this is the first dedicated activity for SCM professionals which has engendered pride in their work.

